# Comparative analysis of death by suicide in Brazil and in the United States: descriptive, cross-sectional time series study: Análise comparativa de morte por suicídio no Brasil e nos Estados Unidos: estudo transversal temporal descritivo

**DOI:** 10.1590/1516-3180.2016.0207091216

**Published:** 2017-04-20

**Authors:** Alexander Abuabara, Allan Abuabara, Carin Albino Luçolli Tonchuk

**Affiliations:** I PhD. Student at the Hazard Reduction & Recovery Center, Department of Landscape Architecture and Urban Planning, Texas A&M University, College Station (TX), United States.; II DDS. Health Auditor, Healthcare Division, Joinville Municipal Authority, Joinville, Santa Catarina (SC), Brazil.; III MD. Health Auditor, Healthcare Division, Joinville Municipal Authority, Joinville, Santa Catarina (SC), Brazil.

**Keywords:** Suicide, Brazil, United States, Public Health, Mental Health

## Abstract

**CONTEXT AND OBJECTIVE:**

The World Health Organization recognizes suicide as a public health priority. Increased knowledge of suicide risk factors is needed in order to be able to adopt effective prevention strategies. The aim of this study was to analyze and compare the association between the Gini coefficient (which is used to measure inequality) and suicide death rates over a 14-year period (2000-2013) in Brazil and in the United States (US). The hypothesis put forward was that reduction of income inequality is accompanied by reduction of suicide rates.

**DESIGN AND SETTING:**

Descriptive cross-sectional time-series study in Brazil and in the US.

**METHODS:**

Population, death and suicide death data were extracted from the DATASUS database in Brazil and from the National Center for Health Statistics in the US. Gini coefficient data were obtained from the World Development Indicators. Time series analysis was performed on Brazilian and American official data regarding the number of deaths caused by suicide between 2000 and 2013 and the Gini coefficients of the two countries. The suicide trends were examined and compared.

**RESULTS:**

Brazil and the US present converging Gini coefficients, mainly due to reduction of inequality in Brazil over the last decade. However, suicide rates are not converging as hypothesized, but are in fact rising in both countries.

**CONCLUSION:**

The hypothesis that reduction of income inequality is accompanied by reduction of suicide rates was not verified.

## RESUMO

**CONTEXTO E OBJETIVO:**

A Organização Mundial da Saúde reconhece o suicídio como uma prioridade de saúde pública. Aumentar o conhecimento dos fatores de risco de suicídio é necessário para se poder adotar estratégias eficazes de prevenção. O objetivo deste estudo foi analisar e comparar a associação do coeficiente de Gini, usado para medir a desigualdade, com as taxas de mortalidade por suicídio em um período de 14 anos (2000-2013) no Brasil e nos Estados Unidos (EUA). Aventou-se a hipótese de que a redução da desigualdade de renda é acompanhada da redução das taxas de suicídio.

**TIPO DE ESTUDO E LOCAL:**

Estudo transversal temporal descritivo realizado no Brasil e nos EUA.

**MÉTODOS:**

Dados populacionais, de óbitos e mortes por suicídio foram extraídos do banco de dados do DATASUS, no Brasil, e do Centro Nacional para Estatísticas de Saúde dos EUA. Dados do índice de Gini foram obtidos dos Indicadores de Desenvolvimento Mundial. Foi realizada análise de séries temporais de dados oficiais do Brasil e dos EUA sobre o número de mortes por suicídio, de 2000 a 2013, e do coeficiente de Gini. As tendências de morte por suicídio foram analisadas e comparadas.

**RESULTADOS:**

Brasil e EUA apresentaram convergência no coeficiente de Gini devida principalmente à redução da desigualdade no Brasil na última década. No entanto, as taxas de suicídio não estão convergindo como foi conjeturado; em verdade, elas estão crescendo em ambos os países.

**CONCLUSÕES:**

A hipótese de que a redução da desigualdade de renda é acompanhada da redução das taxas de suicídio não foi verificada.

## INTRODUCTION

Suicide is a serious public health problem that needs to be faced openly and in a manner that is as well-informed as possible. In fact, there is a need to distinguish between the achieved act (suicide) and suicide attempts (unsuccessful). Suicide is the act of deliberately killing oneself. Over the last 45 years, suicide rates have increased by about 60% worldwide.^1^ Consequently, over this period, suicide has become a public health concern. Over 842,000 people die by suicide every year globally, which is a rate of 11.6 per 100,000 individuals per year, or one death somewhere in the world every 40 seconds. Suicide is the 15^th^ largest cause of death for all age groups in the world.^2^

The World Health Organization (WHO) recognizes suicide as a public health priority. In the Mental Health Action Plan 2013-2020, member states have committed themselves to working towards the global target of reducing the suicide rate by 10% by 2020. The 2014 World Suicide Report “Preventing Suicide: A Global Imperative” aims to increase awareness of the public health significance of suicide and suicide attempts, thereby making suicide prevention a high priority on the public health agenda.^3^

International suicide rates fluctuate between 10 and 15 per 100,000. In some countries, such as Hungary and Korea, the rates reach 21 per 100,000.^4^^,^^5^ In Brazil, the mortality rate due to suicide is considered comparatively low, at 5.8 deaths per 100,000 inhabitants, but it has been increasing among young adults, particularly among males.^5^ Supposedly, social determinants such as decreased income inequality and increased income per capita may have positive associations with decreased suicide rates.^6^ A previous study showed that Rio Grande do Sul has the highest suicide rate among Brazilian states, and it has been suggested that ethnicity, culture, social crises and even the local climate might be factors influencing this.^7^

The suicide rate in the United States (US) is 12.1 deaths per 100,000 inhabitants, and this is the 10^th^ largest general cause of death for all ages.^8^ Approximately 110 Americans die by suicide every day, which means one death every 12.3 minutes, and over 41,000 lives every year.^8^ These numbers represent only the successful suicides: the numbers of suicide attempts are projected to be 20 times higher than this.^9^ Moreover, for every death by suicide there are at least six close individuals whose lives are emotionally, socially and economically severely affected. To increase knowledge of suicide risk factors in specific contexts, effective prevention strategies need to be adopted.^6^

Therefore, the objective of the present study was to ascertain whether income inequality, through the Gini coefficient, is associated with mortality due to suicide, specifically by contrasting Brazil and the US. It was hypothesized that reduction of income inequality would be accompanied by reduction of suicide rates, as suggested in the literature.^6^ The Gini coefficient (World Bank) was selected particularly because it measures the extent to which the distribution of income (or, in some cases, expenditure on consumption) among individuals or households within an economy deviates from perfectly equal distribution.

## OBJECTIVE

This study analyzed and compared the association between the Gini coefficient and suicide death rates over a 14-year period (2000-2013) in Brazil and in the US. It hypothesized that reduction of income inequality would be accompanied by reduction of suicide rates.

## METHODS

This was an exploratory, descriptive and retrospective quantitative study. Brazilian mortality and suicide data from 2000 to 2013 were obtained from the Mortality Information System database (Sistema de Informações sobre Mortalidade, SIM) of the Information Technology Department of the Brazilian National Health System (Departamento de Informática do Sistema Único de Saúde, DATASUS), at <http://www2.datasus.gov.br/DATASUS/index.php>, which was accessed on April 22, 2016. Data on the estimated populations were obtained from the Brazilian Institute for Geography and Statistics (Instituto Brasileiro de Geografia e Estatística, IBGE), at <http://www.sidra.ibge.gov.br/cd/cd2010Serie.asp>, which was accessed on April 22, 2016. The US data were obtained from the Disease Control and Prevention platform (CDC-WONDER), at <http://wonder.cdc.gov/cmf-icd10-archive2013.html>, which was accessed on April 22, 2016, covering the same period of time. Suicide mortality rates in Brazil and the US were analyzed. Gini coefficient data were obtained from the World Bank estimates, at <http://data.worldbank.org/indicator/SI.POV.GINI>, which was accessed on April 22, 2016.

The data were tabulated using Microsoft Excel spreadsheets, which were used to make time series comparisons and evaluations of the data. Subsequently, the figures were plotted. Data covering the period from 2000 (when the new the International Classification of Disease, ICD, 10^th^ revision started to be widely used in the US to represent the causes of death) to 2013 were analyzed, using the following indicators: Gini coefficient variation, number of suicide deaths and number of suicides according to age. Total suicide rates were calculated by dividing the absolute number of deaths due to suicide by the total population of that same year, at the same age, and multiplied by 100,000. Age-adjusted suicide rates were calculated using the direct method, which allowed comparison of rates between the US and Brazil. Adjustment was accomplished by multiplying the age-specific suicide rates by age-specific weights. The age-specific total population of the US was used as a standard population for the Brazilian adjusted rate.

## RESULTS

**Figure 1:**
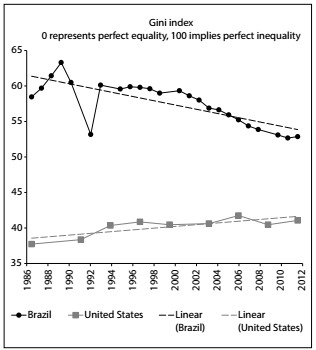
Gini index in Brazil and the United States over the years.

**Figure 2:**
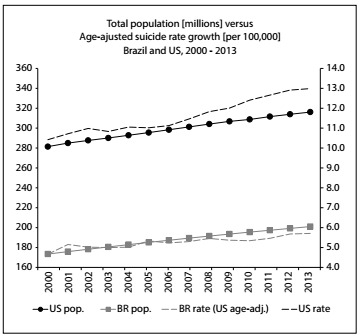
Total population (millions) versus age-adjusted suicide rate growth (per 100,000).

The suicide rate in the US increased by roughly 25%, from 10.4 to 13.0 per 100,000 inhabitants, between 2000 and 2013. In Brazil, the rate of increase after age adjustment in relation to the US population was approximately 22.4%, from 4.7 to 5.7 deaths per 100,000 inhabitants over the same period of time. Table 1 summarizes the populations of Brazil and the US and the numbers of suicides. The last two columns show calculations of suicide rates for each country over the years and the ratio of suicides between the two countries, after age adjustment in relation to the US population. The values in these last two columns grew slightly over the period of time analyzed. Figure 1 shows how the Gini coefficients rapidly converged over time, mainly due to the reduction of inequality in Brazil. Figure 2 shows the age-adjusted ratio of suicides, contrasted with the population growth of the two countries. It can be seen that the numbers of suicides, especially in the US, have been growing in large steps since 2006, both as crude numbers and also as rates.

**Table 1:**
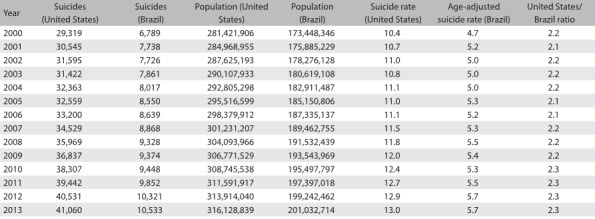
Total number of suicides, population and age-adjusted suicide rate per 100,000 inhabitants and ratio between the countries, over the study period

**Figure 3:**
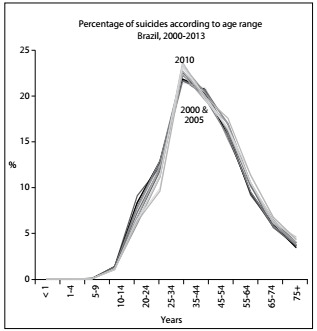
Percentage of suicides distributed according to age range, Brazil, 2000-2013.

**Figure 4:**
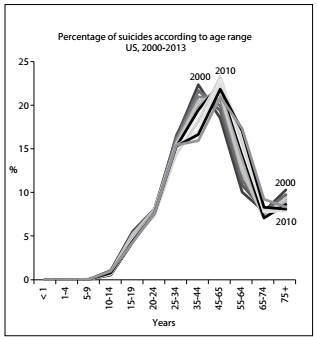
Percentage of suicides distributed according to age range, United States, 2000-2013.

The distributions of deaths due to suicide in Brazil and the US according to age groups over these 14 years are presented in Figures 3 and 4 respectively. A peak at the age range of 25-34 years can be observed in the Brazilian graph, specifically in 2010. This differed from the US, where more suicides occurred at older age ranges, with a peak that moved to older groups: this peak was in the 35-44 group in 2000 and transferred to the 45-54 group over the years.

## DISCUSSION

Brazil is classified as an emerging country, while the US is a developed country. Although the relevance of comparing the Gini coefficient between Brazil and the US may not appear to be scientifically well-founded, this study took into consideration the fact that these countries have converging Gini coefficients, and thus it was hypothesized that some convergence in the numbers of suicides might also be observed. Figure 1 shows how the two countries’ Gini coefficients have been rapidly converging over time, especially because of Brazil’s decreasing inequality, given the significant economic and social changes that have mostly taken place since 1994, with solidification of the democratic regime and attainment of monetary and fiscal equilibrium. Brazil has had superior development due to its thriving economy, which has been reflected in continuous social change. On the other hand, over the period considered, the US faced a serious economic crisis with high unemployment indicators, possibly affecting certain social groups more than others.

Shikida et al.^10^ analyzed how economic variables influence suicide rates, according to Brazilian states. These variables are particularly important among the causes of suicides because they take into account the so-called “contagion effect” that has been described in the literature. In this phenomenon, suicidal behavior triggered by one individual can affect the behavior of other individuals who are living under the same psychological and socioeconomic conditions. These authors established that spatial dependence also seems to characterize suicide data distribution, such that it occurs in all directions, but is inversely related to geographical distance.

According to Durkheim,^11^ the impact of socioeconomic changes, including industrialization, urbanization, secularization, population growth, social integration, migration and female participation in the labor force, has become central to the theories of suicide. In the view of the Durkheimian School, modernization leads to individualism and egoism in relation to the religious system, educational system, economic system and family system; and this erosion of social control reinforces the potential for suicide.^12^^,^^13^^,^^14^ Some studies have found support for Durkheimian theories, such as the positive relationships between suicide and urbanization,^15^ decreasing religiosity and increasing modernization,^16^ population growth^17^ and cultural variables relating to individualism.^18^ Other findings, however, have posed challenges and revisions. Individual factors that affect the risk of suicide include mental disorders, genetics, drug misuse, psychological states and cultural, family and social situations, and some of these frequently coexist.^5^^,^^6^^,^^7^ A correlation between suicidal intent and lethality has also been reported in the literature.^19^

Socioeconomic problems such as unemployment, poverty, homelessness and discrimination may also trigger suicidal behavior.^5^^,^^20^ Suicide is an individual act, although it occurs within the context of a given society, and certain sociodemographic factors, such as gender, age, migration, housing, marital state and occupation, among others, may be associated with it. Brazil’s rapid decline in fertility since the 1960s is the main factor behind the country’s slowing population growth rate, aging population and fast-paced demographic transition.^21^ Consequently, protective factors may have been affected, such as: pregnancy, sense of responsibility towards the family and presence of children in the family.^20^ It is believed that these factors can protect individuals from suicidal behavior, although there is still no scientific evidence to corroborate this assumption.

In most countries, males commit suicide more frequently than females, but the male/female ratio varies from country to country.^5^^,^^19^^,^^22^ Relationships (family and friends) appear to be protective while separation and living alone increase the risk of suicide.^5^^,^^20^ However, these results are not uniform.^19^ The distributions of the deaths due to suicide in Brazil and the US according to age groups over the 14 years studied are shown in Figures 3 and 4, respectively. A peak at the age range of 25-34 years can be observed in the Brazilian graph, in 2010. This differed from the US, where more suicides occurred at older age ranges, with a peak that moved to older groups: this peak was in the 35-44 group in 2000, and transferred to the 45-54 group over the years. According to WHO,^5^ young people are among those most affected but, as found in this study, the numbers may differ between countries.

In Western countries, the typical male-to-female gender ratio for suicide is high: between 2:1 and 4:1.^5^ Data from other studies^23^^,^^24^ confirm that male suicide rates in Brazil and the US are high. Some of the assumed reasons explaining this difference are: higher alcohol abuse among men; men choosing suicide methods of higher lethality; and women coping better with mental illness and thus seeking psychiatric services earlier than men.^23^ Asian countries shows a more balanced ratio, while China is the only country in the world where the suicide rate among women is higher than among men. Social and cultural factors may provide explanations for China’s high female suicide rates.^25^^,^^26^

Santa Catarina and Rio Grande do Sul are Brazilian states that stand out regarding socioeconomic development rates. However, they present critical suicide rates, above the Brazilian average.^7^^,^^27^ Studies have shown that a direct relationship between economic development and suicide rates cannot be easily established. However, Machado et al.^6^ and Kim^28^ concluded that inequality is an important determinant of suicide, both in Brazil and in the US. These studies found linkages between state-level income inequality and the risks of dying due to suicide. Future research should address smaller demographic areas and compare indexes other than the Gini, or even aggregate them with additional healthcare system data measurements, and contrast the combination of such data with suicides and homicides.

It is important to bear in mind that the main racial groups in the US are classified merely as black and white, whereas in Brazil, in addition to these two groups, large numbers of people are grouped as *pardo* (brown/mulatto). Thus, the race categorizations of these two countries are not directly comparable. However, independently of each other, they respect the current census groupings and it is noteworthy that, internally, there are important differences between the rates that can be explored in future research.

Another limitation that it is important to highlight relates to the locations where suicides occurred. It has been suggested in the literature^29^^,^^30^^,^^31^ that differences between rural and urban areas are significant. Further studies using supplementary sociodemographic variables could be conducted to cover these possibilities. Bando et al.^23^ observed that in addition to the higher risk of suicide among singles, divorcees and widowers, compared with married people, foreigners were also at higher risk of suicide. Immigrant refugees are more likely to present serious mental disorders and, thus, commit suicide. Social disadvantage is another putative reason for explaining the higher risk of suicide among immigrants.^23^ Lester^32^ identified associations between quality of life and rates of personal violence that were only valid for some groups: in particular, for whites and blacks in the US. Prevention programs and strategies need to take these factors into account. Epidemiological analysis is an important tool for identifying population subgroups that are at increased risk of suicide, thus helping to develop prevention and sentinel strategies for high-risk groups.

Common sense suggests that people should become happier as their conditions of life improve. If poverty and forms of oppression such as sexism and racism could be eliminated, if the environment could be cleaned up, and if education and cultural offerings for people could be improved, then society ought to be considerably happier. Unfortunately, one sociological theory predicts the opposite of this. Henry and Short^33^ argued that if people have clear external sources that they can blame for failures and unhappiness, then they will feel angry and be outwardly aggressive. In contrast, if there is no clear external source of blame, then people hold themselves responsible. In this case, individuals are more likely to feel depressed and culpable, thus increasing the chance that they might kill themselves. There is evidence to support this idea. In both the United States and South Africa, for example, the oppressed (blacks) have higher homicide rates, and the oppressors (whites) have higher suicide rates.^32^ Lester^34^ found that the states in the US with the highest quality of life (rated on a variety of aspects) had the highest suicide rates and the lowest homicide rates. Similarly, in a study on various countries, Lester^35^ found that countries with the highest quality of life had the highest suicide rates and the lowest homicide rates. It appears that if life is better, suicide becomes more common and homicide less prevalent.

While the Gini coefficients of Brazil and the US are converging over time, suicide rates are simply growing in both countries (Figure 2). Conversely, it seems plausible to extend the sociological theory presented by Henry and Short^33^ to make comparisons between the low suicide rates in Brazil and the higher rates in the US.

## CONCLUSION

Based on the fact that Brazil and the US have converging Gini coefficients, with reduction of inequality in Brazil and slightly increased inequality in the US, this study hypothesized that this reduction of income inequality would extend to reduction of suicide rates. However, despite convergent Gini coefficients, suicide rates are growing in both countries. The supposition that reduction of income inequality would be accompanied by reduction of suicide rates was not verified.
